# The intergenerational succession and financialization of Chinese family enterprises: Considering the influence of heirs’ growing experience

**DOI:** 10.3389/fpsyg.2022.1004997

**Published:** 2022-10-10

**Authors:** Shengchao Ye, Wei Wang, Yidong Li, Haohan Wang, Xinmiao Zhou

**Affiliations:** ^1^Business School, Ningbo University, Ningbo, Zhejiang, China; ^2^School of Business Administration, Henan University of Economics and Law, Zhengzhou, Henan, China; ^3^School of Finance, Zhejiang Gongshang University, Hangzhou, Zhejiang, China

**Keywords:** Chinese family enterprises, intergenerational succession, financialization, growing experience, mixed-methods

## Abstract

As a mixed-methods research in economics and psychology, this study aimed to analyze the influence from the intergenerational succession on the financialization level including asset financialization and revenue financialization, and further test the moderating effect of the heirs’ typical growing experience according to The Imprinting Theory, based on the 2009–2020 annual data of listed family enterprises of China. There were two key findings. First, the effect of Chinese family enterprises’ intergenerational succession on asset financialization was positively significant while the effect on revenue financialization was not significant, indicating that the financialization behavior has not brought about effective financial profits. Second, among the heirs’ typical growing experiences, their parents’ entrepreneurial experience during their childhood, oversea study experience, and MBA education experience had the significantly positive moderating effects on the influence from intergeneration succession to asset financialization level of Chinese family enterprises, which was an important internal mechanism for the heirs to promote the financialization process of family enterprises.

## Introduction

The family enterprise is the main component of China’s private economy. In the rapid development of China’s economy in the past 40 years, family enterprises and their first generation of entrepreneurs have been playing a key role. Most of Chinese family enterprises were founded in the 1980s. With the aging of the first entrepreneur generation, Chinese family enterprises have been generally moving into the intergenerational succession stage, which is becoming more and more urgent in recent years (Xu et al., 2019). Abundant studies demonstrated that the intergenerational succession of management power influenced a lot on the operation of family business, and mainly negative ones (Zhu and Lv, 2019; Chen et al., 2021; Li X. et al., 2021). Since the 2008 global financial crisis, Chinese family enterprises have reached the peak of intergenerational succession, and China’s macro economy has meanwhile gradually entered a “new normal,” with shrinking market demand and worsening overcapacity in the real economy (Tang and Zhang, 2019). Therefore, many enterprises are facing great challenges to their original development mode with their main business as the core, and then the financial asset allocation has become the main choice for them to maintain business performance (Wang, 2018; Feng et al., 2022). The financialization of enterprises is the basis of macroeconomic transition from real to virtual. Many researchers were obsessed with discussing its causes and influences from the internal and external aspects.

For many Chinese family enterprises, they are facing the combination of internal intergenerational succession with the strengthening trend of macroeconomic financialisation. Undoubtedly, this is a critical period for their long-term development. According to the theory of authority, heirs of family enterprises will generally suffer the embarrassment of insufficient legitimacy and role crisis caused by lack of experience (Li et al., 2015). Facing this dilemma, one of the core coping ideas of heirs is to create profits much more quickly by opening up “New Territories,” including developing new businesses outside the main business, reforming corporate strategy, trying to diversify investment, etc. (Cai et al., 2019; Zhu and Lv, 2019; Porfírio et al., 2020). Unfortunately, the outcome did not meet their expectations. In view of the above findings, promoting the financialization of enterprises seems to be an excellent option for heirs to acquire the potentially high profits from the financial market with huge appeal. However, researches on the relationship of intergenerational succession and financialization of Chinese family enterprises have rarely been reported.

The psychological characteristics of leaders, such as cognitive mode and value orientation, are particularly essential for the strategic choice and decision-making of family enterprises (Hu and Wu, 2016). The different psychological characteristic between heirs and founders is the main reason for the multiple impacts of intergenerational succession on family enterprises. The Imprinting Theory can help to interpret the mechanism behind this phenomenon. According to this theory, the growing experience of the heir is a key factor. The “imprinting” formed in specific sensitive periods for adapting to the environment, which is also named as cognitive mindset and way of thinking, will have a continuous impact on their future management style and decision-making tendency (Dai et al., 2016). Similar evidences have been reported in other studies (Yu et al., 2020; Zhang and Luo, 2021).

Will Chinese family enterprise heirs, whose “imprinting” are obviously different from their parents (Li W. et al., 2021), tend to make riskier and speculative decisions and as a result embrace the macro trend from real economy to financial market to promote the financialization of enterprises more aggressively? This is the key question of our research. Experiences in the sensitive period for shaping the heirs’ psychological characteristics will help us understand the behavioral tendencies and mechanisms of family business heirs (Ahrens et al., 2019; Li et al., 2020).

Based on the information summarized above, the study explored the influence from the intergenerational succession on the financialization of Chinese family enterprises. Referring to previous studies, we, respectively, constructed two indicators of asset financialization and revenue financialization, so as to examine the behavior and effect of heirs promoting the financialization of family enterprises. Furthermore, this study took the heirs’ typical growing experiences which had great impacts on their psychological development and cognitive mindset into consideration (Pan et al., 2018; Sarfraz et al., 2022), elucidating the mechanism of intergenerational succession on family enterprises financialization. Notably, a mixed-methods research of economics and psychology was applied.

The remaining part of this paper was arranged as follows: The second part was “literature review and research hypotheses.” This part reviewed the literature on intergenerational succession of family enterprises, financialization of enterprises and the growth experience of heirs, and further put forward research hypotheses on this basis. The third part was “research design,” which presented the research data, main variables and research methods. The third section was “results,” which present the empirical results on the influence of generational succession of family enterprises on their financialization level. On this basis, we further analyze the moderating effect of heirs’ growth experiences. The last part was “discussion,” which further expanded the analysis of the empirical results and prospected the future research directions.

## Literature review and research hypothesis

### Family enterprises’ intergenerational succession

The core goal of family business is to realize the intergenerational succession of family wealth and the sustainable development of their enterprises (Miroshnychenko et al., 2021). Under the guidance of this goal, the particularity of family business behavior and the influence of intergenerational succession on family business have attracted many scholars’ attention for a long time. Summarized from the existing literature about the intergenerational succession of family enterprises, it was found that due to the early starting of family business in west countries, western researchers did the earliest research in this field and have established a series of relevant research topics including the impact of intergenerational succession on corporate performances (Bennedsen et al., 2005b; Bertrand et al., 2008; Cucculelli, 2009) and business behavior of enterprises (Bennedsen et al., 2005b; Villalonga et al., 2006), the impact of characteristics of founders on the intergenerational succession of family enterprises (Goldberg et al., 1993; Davis et al., 2001), the successor training strategy of family enterprises (Bennedsen et al., 2005a; Cucculelli, 2009) and so on.

Chinese family enterprises are generally established for a short period, their early development mode is relatively extensive (Li X. et al., 2021). They were more dependent on leaders because of the lack of standardized formal management system, and a mature business environment including an effective professional manager market has not been formed in China (Xu et al., 2019). In addition, China has been experiencing a critical economic transformation, and enterprises are facing many new challenges in this process. The influence of intergenerational succession on Chinese family enterprises is particularly striking. In recent years, with Chinese family enterprises entering the succession stage gradually, Chinese scholars have been increasingly enriching their research achievements in this field. The achievements have not only covered traditional research topics (Cai et al., 2019; Weng and Chi, 2019; Zhang and Luo, 2021), but also extended this research field based on the particularity of internal and external conditions faced by Chinese enterprises, such as CSR of intergenerational succession in family enterprises (Chen, 2018), operational strategic capital (Bin-Feng et al., 2022), investment in enterprise innovation (Xiong et al., 2021) and so on. Moreover, mechanisms of more factors are further elucidated, including family board’s excessing control (Liu et al., 2021), heirs’ education experience (Zhu and Lv, 2019), first entrepreneurs’ altruism (Zhu et al., 2021), and intergenerational transmission of political resources (Yang et al., 2022).

In conclusion, the researches of family enterprise intergenerational succession are obviously influenced by the representative issues of the times. Besides the core index of intergenerational succession, the research topics are often closely related to the macro environment of enterprises. To a certain extent, exploring the particularity of family enterprises in the general features of macro economy is an inevitable way to investigate and deepen this research area. The booming trend of financialization of enterprises under the current “new normal” of China’s economy is an important background for the intergenerational succession of Chinese family enterprises (Wang et al., 2017). Financialization is undoubtedly the decision focus that the heirs cannot neglect when adjusting their enterprise strategy. Out of a need to validate their own legitimacy (Li et al., 2015), build their competence authority (Huang et al., 2018), and fulfill their own values (Wang et al., 2021), will the heirs be more inclined to take financialization as a way or choice to adjust their corporate strategy to achieve the goals mentioned above with a higher rate of return in a shorter term? Until now, researchers have not given a complete explanation.

### Financialization of enterprises

The academia’s attention to financialization first started from macroeconomy. Researchers believed that the over-financialization of economy was one of the most important reasons for the American subprime mortgage crisis in 2007 (Duchin et al., 2009; Peng et al., 2018). The financialization of the macro economy was often accompanied by the constant increases of the size, status and influence of the financial sector, which in turn led to the increasing proportion of profits from financial speculation and investment activities in GDP (Krippner, 2005). Since the financialization of enterprise is the basis of economic financialization, the relevant researches on it possess great importance for preventing economic over-financialization and hence have aroused broad attention over recent years. From the current viewpoints, the financialization of enterprises can be defined with two aspects: behavior and result (Peng et al., 2018; Cai et al., 2019; Zhao and Su, 2022). From the perspective of behavior, financialization is a resource allocation method adopted by enterprises focusing on capital operation rather than traditional main business. As for the result aspect, the financialization of enterprises means that the principal revenues of enterprises come from financial activities rather than main business (Liu et al., 2018). When measuring the two aspects, the financial analysis framework proposed by Penman and Yehuda (2009) has been applied in most studies, dividing corporate asset/revenue into financial asset/revenue and operating asset/revenue. Therefore, the ratio of financial assets to total assets represents the behavior aspect, while the ratio of financial revenue to total revenue represents the result aspect (Song and Yang, 2015; Du et al., 2019). In the following, we refer to them as asset financialization and revenue financialization, respectively.

With China’s economic financialization attracting more and more interests, abundant studies have been carried out in this field, including two categories: the causes and the effects of enterprises’ financialization. Among the various reasons for enterprises’ financialization, the critical role of leaders attracted our attention. Leaders’ confidence level, authority, work experience, academic experience, and other factors will significantly affect the level of enterprises’ financialization (Yan and Chen, 2018; Du et al., 2019; Ke et al., 2019). The Imprinting Theory can provide a convincing explanation for these relationships. As for the impact of corporate financialization, the “crowding-out effect” and the “reservoir effect” reveal the different performances of different financial motivations on the resource allocation of enterprises. If enterprises allocate financial assets with “precautionary” purpose, the “reservoir effect” comes into play. In this case, enterprises generally increase asset liquidity by financial asset allocation to ensure the sufficient capital for main business investment, which would therefore not been “replaced” by the financial assets. If enterprises allocate financial assets for profit purpose, the “crowding-out effect” comes into play. In this case, enterprises intend to obtain higher profits from financial investments rather than the main business, which would ultimately been “replaced” by financial assets (Hu et al., 2017; Xun et al., 2017). A large number of studies have shown that the profit purpose is the main purpose when allocating financial assets in Chinese enterprises (Qi et al., 2021; Su and Liu, 2021; Xu and Xuan, 2021).

Most of the main businesses of Chinese family enterprises belong to the real economy. Due to the shrinking market demand and excess capacity, the profits from the main business are constantly compressed, and the limitations of old business strategy are increasingly obvious. Owing to the long cycle, high uncertainty and high risk of failure of the main business investment, it is quite difficult for the heirs of family enterprises to achieve rapid profits and establish their authority through the main business (Su and Liu, 2021). Aiming to establish their own legitimacy, authority and self-value, they are more likely to make profits quickly through financialization, thus showing the “crowding out” of financial assets on the main business assets. However, theoretically, the return on operating assets could hardly been affected by the allocation ratio of financial assets (Tang and Zhang, 2019). The imperfection and unsoundness of China’s financial market environment would result in the relative scarcity of financial assets with investment value. Their poor record in other areas also hardly inspires confidence that they will succeed in a more unfamiliar field. If the heirs increase their holdings of financial assets, there is the strong possibility that they underestimate the risks of financial markets (Du et al., 2019), leading to the failure.

Based on the thorough analysis of family enterprise intergenerational succession and enterprises’ financialization, two hypotheses are proposed as follows:

*H1*: intergenerational succession has a significantly positive effect on the asset financialization level of Chinese family enterprises.

*H2*: intergenerational succession has no obvious effect on the revenue financialization level of Chinese family enterprises.

### The heirs’ growing experience

As the description of Upper Echelons Theory, the cognitive structure and personal values of enterprise managers are the important basis of their decision-making behavior, and their personal decision-making style will have an important impact on the strategic choice and organizational performance of enterprises (Hambrick et al., 1998). Because of the “absence” of formal institutions in Chinese enterprises, the informal institutions, such as the personal ability, value orientation, and social relations of enterprise managers, appear more important, especially for family enterprises (Marquis and Tilcsik, 2013). It is not surprising that the difference between heirs and their parents has become one of the focuses in the field of intergenerational succession in family enterprise. This difference is particularly stark in China, where the generation gap has been highlighted by the rapid development in the past 40 years (Li et al., 2020). According to the general law of individual psychological development, the differences in decision-making styles and tendencies between the heirs and their parent founders of family enterprises are largely derived from the behavior and thinking inertia shaped by different growing experiences, thus forming different risk preferences and management styles, and ultimately leading to the change of business strategy (Bernile et al., 2017; Ishfaq et al., 2020).

What experience will significantly cause the psychological differences between heirs and their parents? According to The Imprinting Theory, only the strong matching imprinting formed in the sensitive period of individual growth has a sustained impact on their behaviors (Pieper et al., 2015). Among various important growing experiences, it is generally believed that the early growing environment, education experience and early working experience especially affect and shape the heir’s psychological reaction mechanism at different stages, and finally reflect in the heir’s specific management behavior (Khan et al., 2021; O’Sullivan et al., 2021). The four types of growing experiences received most research attention: (a) parents’ entrepreneurial experience during the heir’s childhood; (b) overseas study experience; (c) MBA education experience; and (d) working experience outside the family enterprises.

For experience (a), some studies found that if the heir has experienced the hard entrepreneurial process of his parents in his/her childhood, he/she will have a sense of rejection of the enterprise (Cai et al., 2019). After inheriting the family business, they are more inclined to weaken the main business, pursue short-term profits, and even substantially change the corporate strategy without any psychological burden (Leve et al., 2010; Cai et al., 2019). Other studies found that heirs who witnessed their parents’ entrepreneurship in childhood will form psychological traits of self-confidence and independence by internalizing their parents’ behavior patterns, and then tend to prove themselves in new fields rather than follow the original business of the family enterprises (Carr and Sequeira, 2007; Wyrwich, 2015).

For experience (b), heirs who have studied abroad experienced the collision of Eastern and Western cultures in early adulthood, which is quite profound (Zhu and Lv, 2019). The cross-cultural learning experience made them more confident in adapting to contemporary Western business norms (Bai et al., 2020). They are more familiar with contemporary Western capital operations represented by financial markets, and their decision-making style has a higher risk preference (Levin and Barnard, 2013). However, it is more difficult for them to inherit the invisible assets of the founders and lack of authority, making it difficult for them to develop the original business of the enterprise. Therefore, they are more likely to adjust the business structure of the enterprise and develop new areas (Giannetti et al., 2015).

For experience (c), the main purpose of MBA education for business managers is to improve their management ability and accumulate business contacts (Li et al., 2016). However, some researchers have observed a strong correlation between the richness of managers’ business knowledge and their risk preferences (Faleye et al., 2014). Short-term profit-seeking behaviors are favored, such as business mergers, acquisitions, and financial investment (Miller and Xu, 2016). And the peer effect formed by social networks further amplifies this trend (Liu et al., 2015).

For experience (d), early career experience has a profound impact on individuals, because it makes them aware of workplace norms and standards during the sensitive period of identity transformation, thus continuously influencing their subsequent career (Du et al., 2019; Li W. et al., 2021). Working experience in different industries makes the business vision and contacts of managers much broader, and even renders managers overconfident (Malmendier et al., 2011). Some studies have shown that heirs with work experience outside the family business are more inclined to “make quick money” through real estate investment, financial investment, and other ways (Dai et al., 2016). Of course, the conflict of different corporate cultures will also make it difficult for heirs to accept the existing business model, pushing them to find a new industry that could satisfy them (Custódio and Metzger, 2014).

Based on the logical analysis of heirs’ growing experience and its potential influences, four hypotheses are further put forward as follows:

*H3*: parents’ entrepreneurial experience during the heir’s childhood has a significantly positive moderating effect on the family enterprise’s financialization.

*H4*: the heir’s oversea study experience has a significantly moderating positive effect on the family enterprise’s financialization.

*H5*: the heir’s MBA study experience has a significantly positive moderating effect on the family enterprise’s financialization.

*H6*: the heir’s working experience outside the family enterprise has a significantly moderating positive effect on the family enterprise’s financialization.

## Research design

### Sample and data

This study took family enterprises listed in China’s A-share market from 2009 to 2020 as the research object. The reason to choose data starting from 2009 is that Chinese listed companies are required to disclose complete financial asset data after 2008. The data was collected mainly from CSMAR and Wind databases. The family enterprise is defined as an enterprise in which the actual controller can be traced back to the founding family and the final controller is directly or indirectly the largest shareholder of the listed company (Bennedsen et al., 2005a). In addition, by the aid of the “senior executive’s personal information,” annual report, listing announcement, prospectus and other information of these listed companies, the information of those family enterprises which have finished the intergenerational succession and the heirs’ personal information were confirmed. Further, information about the heirs’ growing experience was manually collected and verified with the “Tianyancha search,” “Baidu Encyclopedia,” “Sina Finance” and other channels. Based on the original samples, data in this study was cleaned and processed as follows, referring to the mainstream practices of other researchers (Cai et al., 2019). First, samples of enterprises with incomplete key variables values are eliminated. Second, samples of financial and insurance enterprises are eliminated. Third, samples of enterprises labeled with ST or *ST during succession are eliminated. All macroeconomic data was acquired from National Bureau of Statistics of China.

Based on data collection and cleaning, a total of 20,299 groups of observational data of annual enterprise information were obtained, covering 2078 listed family enterprises. The succession of the chairman or CEO was regarded as a symbol of the finalization of the sample for intergenerational succession enterprises, according to existing literature (Cucculelli, 2009). Taking this as the criterion, 158 family enterprises that have completed succession in 2009–2020 were determined, with 1,392 groups of observation values.

### Variables

#### Dependent variables

The financialization level of Chinese family enterprise was chosen as the dependent variable, described with two indexes, i.e., asset financialization level (FS) and revenue financialization level (FR). The asset financialization (FS) measured the financialization level of Chinese family enterprises from the perspective of behavior (capital input), which can be calculated by the ratio of corporate financial assets to the total assets of the company. Financial assets consist of trading financial assets, derivative financial assets, financial assets available for sale, hold-to-maturity investment, investment real estate and equity of financial institutions in long-term equity investment (Du et al., 2017; Wu et al., 2021). Revenue financialization level (FR) measured the financialization degree of Chinese family enterprises from the perspective of result (investment income), which is calculated by the ratio of corporate income from financial channels to the total revenue of enterprises (Zhang and Zhang, 2016; Yang et al., 2019). The more comprehensive evaluation of the financial level of enterprises was achieved with the combination of these two dimensions.

#### Independent variables

The independent variable in this paper was “whether the family enterprise had completed intergenerational succession.” Referring to the data, there was monthly difference for the enterprises that complete the intergenerational succession in the same year. Considering that the data applied in this study was annual data, and there were still big limitations on the role that the heirs can play in the family business at the first year of the succession, the independent variable was set as “1” if the completion of the intergenerational succession reached or exceeded one year, otherwise it was set as “0.”

#### Moderator variables

The moderator variables involved in this paper was the heirs’ typical growing experience which was measured from the following aspects: a. parents’ entrepreneurial experience during the heir’s childhood (Childhood); b. overseas study experience (Overseas); c. MBA education experience (MBA); d. working experience outside the family enterprises (Outjob).

#### Control variables

Referring to the general practice of relevant literature (Anderson et al., 2012) all control variables were classed into three parts in this study. The first part was the control variables of enterprise characteristics, including company size, asset-liability ratio, net profit margin of assets, main business growth rate, capital investment scale, etc. The second part was macroeconomic control variables, including national GDP growth rate, money supply growth rate, loan interest rate, etc. The third part was variables of the heirs’ personal characteristics, including gender, age of succession, education level, cognitive difference with parents, etc.

All variables involved in this paper and their detailed meanings are shown in Table 1.

**Table 1:** Descriptions for the main variables.

Type of variables	Name of variables	Abbreviation	Description
Dependent variable	Asset financialization level	FS	The ratio of financial assets to the total assets of the enterprises, equals to financial assets/total assets in the end of the year.
	Revenue financialization level	FR	The ratio of revenue from financial channels to the total revenue of enterprises
Independent variable	Family enterprise’ intergenerational succession	Post	1 for one year after the heir’s succession, otherwise 0.
Moderating variables	The heir’s childhood experience	Childhood	Whether the heir’s childhood (5–15 years old) experienced the initial stage of the family enterprise, yes valued 1; no valued 0. (Bernile et al., 2017)
	The heir’s oversea study experience	Overseas	Whether the heir has oversea study experience yes valued 1, no valued 0
	The heir’s MBA education experience	MBA	Whether the heir has MBA or eMBA education experience, yes valued 1, no valued 0
	The heir’s working experience outside the family enterprise	Outjob	Whether the heir has the working experience outside the family enterprise, yes valued 1, no valued 0
Control variables	Company size	Lnsize	Take the logarithm of total assets at the end of the period
	Asset-liability ratio	Lev	Ratio of total liabilities to total assets at the end of the period
	Net profit margin on assets	ROA	Ratio of annual net profit to total assets at the end of the period
	Operational cash flow	CFO	The ratio of ending operating cash flow to ending total assets
	Main business growth rate	MOIG	Equal to (current year main business income–last year main business income)/last year main business income
	Scale of capital investment	Invest	Ratio of capital investment to total assets at the end of the period
	GDP rate of increase	GDP	The annual growth rate of the macro economy*100
	Growth rate of money supply	M2	The money supply of the current year/the money supply of the previous year −1
	Loan rate level	Loanrate	One-year bank loan rate
	Age of succession	Age	The age of the heir when he/she take over the enterprise
	Working time in the family enterprise	Train	The time period when the heir working in the family enterprise
	Cognitive difference with the parent founder	Cogdiff	The heir’s education minus parental education
	Gender of the heir	Sex	Gender of the heir
	Degree of the heir	Degree	The successor’s highest educational degree

### Methods

The key content in this study is the impact of family enterprise intergenerational succession on the level of financialization and the moderating role of the successor’s growing experience. The mixed-methods was applied in this study including modified Propensity Score Matching (PSM), Difference in Difference (DID) and Moderating Effect Test Model. The reason for the choice of these methods can be summarized as followed. If the intergenerational succession of a family enterprise can be regarded as a natural or quasi-natural experiment, the DID method can be used to compare the financialization level of the family enterprises that have completed the intergenerational succession (the treatment group) with that of the family enterprises that have not completed the intergenerational succession (the control group), and the processing effect of the intergenerational succession can be obtained. However, intergenerational succession is not a completely exogenous random event. First, besides the choice of succession, there are other segment that must be completed, such as choices of professional managers and other shareholders, etc. The completion of intergenerational succession of family enterprises is the final result of investigation and game for a long period. Second, intergenerational succession is only one of many factors affecting the process of enterprise financialization. And the difference in the level of financialization among different enterprises may be caused by other factors that cannot be observed or do not change over time. In a word, direct application of the DID method for comparison may produce certain deviations.

The combination of modified PSM and DID methods was applied in this study to gain results more credible and accurate. First, the probability of being at the financialization level of a certain enterprise in the same year was taken as the explained variable, and then a series of enterprise characteristic variables were matched to search the control group whose family enterprises have completed the intergenerational succession to eliminate the problem of sample selection, with PSM. After gathering samples of the treatment group and the control group, the DID estimator independent of linear regression which represent the real effect of the intergenerational succession on the financialization of Chinese family enterprises is obtained with DID method, thus ensuring the maximum accuracy of the estimation results.

After determining the relation between the intergenerational succession and the financialization level of family enterprise, the moderating effect of the heir’s growing experience was further tested with Moderating Effect Test Model, to help us to understand this relation more comprehensive in the aspect of heir’s individual psychological development.

#### Propensity score matching

The basic idea of PSM is to find the matching enterprises closest to the enterprises in the treatment group. In brief, the matching enterprises’ characteristics are the closest to the financialization of enterprises, apart from the completion of intergenerational succession. The matching enterprises were taken as the control group, avoiding the selection bias caused by the statistical characteristics of the sample enterprises themselves.

Considering the fact that intergenerational succession of family enterprises happens every year during the probationary period, the year-by-year PSM was adopted to conduct the one-to-one nearest neighbor matching without putting back for each enterprise that has completed the intergenerational succession, referring to the mature practices of other researchers (Greenaway et al., 2005; Heyman et al., 2006). The basic form of PSM method can be expressed as the following equation:


(1) $$p\left(X_{i}\right)\text{≡}P_{r}\left(D_{i}=1|X_{i}\right)$$

Where, $D=\left\{0,1\right\}$, means if it belongs to the treatment group, $X_{i}$ means covariable vector.

In this study, year-by-year PSM can be explicated as follows:


(2) $$P_{\mathrm{it}}\left(\mathrm{Fin}\right)=\alpha_{\mathrm{it}}+\sum \beta_{\mathrm{it}}\cdot \mathrm{Cova}r_{\mathrm{it}}+\varepsilon_{\mathrm{it}}$$

Where, i represents enterprises, t represents the year, $P_{\mathrm{it}}$ the probability of financialization of the enterprise i in t years, $\mathrm{Cova}r_{\mathrm{it}}$ is a set of matching variables, representing a set of factors associated with the financialization of an enterprise.

#### Difference in difference

DID can supplement and correct the unavoidable omission of control variables in PSM (Dehejia and Wahba, 1998). DID is a two-way fixed effect regression model in essence, and its basic form can be expressed as the following equation:


(3) $$Y_{\mathrm{it}}=\alpha_{0}+\alpha_{1}T_{\mathrm{it}}+\beta X_{\mathrm{it}}+\delta_{i}+\mu_{t}+\varepsilon_{\mathrm{it}}$$

where, i represents the enterprise, t represents the year, $Y_{\mathrm{it}}$ is the dependent variable, $T_{\mathrm{it}}$ is the treatment variable, $X_{\mathrm{it}}$ represent other control variables, $\delta_{i}$ is entity fixed effects, $\mu_{t}$ is time fixed effect, $\varepsilon_{\mathrm{it}}$ is random disturbance. While specifying to this study, the multiple DID can be expressed as follows:


(4) $${\mathrm{Fin}}_{\mathrm{it}}=\alpha_{0}+\alpha_{1}{\mathrm{Treatment}}_{i}\cdot {\mathrm{Post}}_{\mathrm{it}}+\sum \alpha_{j}\cdot X_{jit}+\delta_{i}+\mu_{t}+\varepsilon_{\mathrm{it}}$$

where, ${\mathrm{Fin}}_{\mathrm{it}}$ represents the financialization level of the enterprise i in t years, $X_{jit}$ represents the control variables affecting the level of enterprise financialization. $\mathrm{Treatmen}t_{i}$ indicates whether the enterprise belongs to the processing group, ${\mathrm{Post}}_{\mathrm{it}}$ representative enterprise i whether finish the intergenerational succession in t, ${\mathrm{Treatment}}_{i}\cdot {\mathrm{Post}}_{\mathrm{it}}$ is the DID statistic, and also is the core variable of this study. If the coefficient $\alpha_{1}$ is significant, it means intergenerational succession has a significant impact on the financialization level of family enterprises.

#### Moderating effect test model

In order to further test the role of the heir’s growing experience in the relationship between family enterprise’s intergenerational succession and the enterprise’s financialization level, this paper set the following moderating effect test model:


(5) $$\begin{array}{c} {\mathrm{Fin}}_{\mathrm{it}}=\gamma_{0}+\gamma_{1}{\mathrm{DID}}_{\mathrm{it}}+\gamma_{2}{\mathrm{MOD}}_{\mathrm{it}}+\gamma_{3}{\mathrm{DID}}_{\mathrm{it}}\cdot {\mathrm{MOD}}_{\mathrm{it}}\\ \;+\sum \gamma_{j}\cdot X_{jit}+\delta_{i}+\mu_{t}+\varepsilon_{\mathrm{it}} \end{array}$$

Where, ${\mathrm{MOD}}_{\mathrm{it}}$ is moderating variable. If the coefficient $\gamma_{3}$ of the cross term ${\mathrm{DID}}_{\mathrm{it}}\cdot {\mathrm{MOD}}_{\mathrm{it}}$ is significant, the heir’s growing experience plays a moderating role in the financialization level of the family enterprise.

## Results

### Results for year-by-year PSM

Referring to the method in classic literature (Dehejia and Wahba, 1998), the Logit probability model was taken to estimate the conditional probability in this study. Following the method of Yang et al. (2019), seven observable matching variables were used to calculate the probability of each enterprise in the certain point of financialization level at current year and then the enterprises in the treatment group and control group were matched. Specifically, the seven observable matching variables are logarithm of enterprise company size (Lnsize), asset-liability ratio (Lev), net profit margin on assets (ROA), cash flow operation (CFO), main business growth rate (MOIG), scale of capital investment (Invest) as well as industry attributes as dummy variables.

The descriptive statistics of variables in the whole sample after matching are listed in Table 2, containing 1,392 observation data in the final treatment group and the control group, respectively. In the process of matching, the balance hypothesis of score matching in each year is tested to verify the reliability of matching results in this study. Only the inspection results for 2017 is displayed here (Table 3), showing the difference between the control group and the treatment group before and after year-by-year PSM. It shows that the standard deviation of most matching variables is basically controlled within 10% after matching, and the standard deviation is significantly reduced after year-by-year PSM than before matching. In addition, the *t* statistics of all variables after year-by-year PSM are not significant. The matching results of other years (data not shown) are similar to that of 2017. It is indicated that there is no significant difference in matching variables between the treatment group and control group, which means, whether it belongs to the treatment group is independent of the matching variables in the case of a given propensity score $P\left(X\right)$. Therefore, the matched samples in this paper meet the common trend assumption.

**Table 2:** Descriptive statistics of variables after year-by-year propensity score matching (PSM).

Variable	Obs	Mean	Std. Dev.	Min	Max
FS	2,784	0.043	0.092	0.000	0.981
FR	2,784	0.021	0.115	−1.477	3.153
Lnsize	2,784	9.497	0.481	7.501	11.665
LEV	2,784	0.416	0.271	0.008	6.348
ROA	2,784	0.042	0.088	−1.577	0.817
CFO	2,784	1.284	3.898	−0.645	89.187
MOIG	2,784	1.213	21.021	−29.476	961.349
Invest	2,784	0.053	0.051	0.000	0.426
GDP	2,784	7.577	1.295	6.100	10.600
M2	2,784	12.805	5.063	6.991	28.423
Loanrate	2,784	5.095	0.821	4.350	6.560

**Table 3:** Results of balance hypothesis test.

Variable		Mean		Bias	–Bias	*t*	*t*-test
		Treated	Control	%	%		*p* > *t*
Lnsize	Unmatched	9.650	9.490	33.5	84.9	3.96	0.000
	Matched	9.650	9.674	−5.1		−0.45	0.653
Lev	Unmatched	0.407	0.367	18.3	89.1	2.47	0.014
	Matched	0.407	0.402	2.0		0.18	0.857
ROA	Unmatched	0.277	−0.050	−18.5	16.5	−3.03	0.002
	Matched	0.277	0.046	−15.5		−1.48	0.141
CFO	Unmatched	0.856	1.094	−12.4	20.9	−1.23	0.219
	Matched	0.856	0.666	9.8		1.43	0.155
MOIG	Unmatched	1.295	0.511	8.5	51.1	2.10	0.036
	Matched	1.295	1.678	−4.2		−0.30	0.766
Investrate	Unmatched	0.044	0.479	−8.8	62.9	−1.05	0.94
	Matched	0.044	0.453	−3.3		−0.31	1.0

### Results for multiple DID

Based on the sample data of treatment group and control group after year-by-year PSM, the multiple DID analyses were further carried out to investigate the influence of intergenerational succession on asset financialization level and revenue financialization level, respectively. In order to eliminate the possible interferences of other unobserved variables which do not change over time, the dual fixed effect model is adopted for estimation and the results are shown in Table 4. Based on the principle of DID, the coefficient $\alpha_{1}$ of the cross-term Treatment × post, reflecting the change of the financialization level of family enterprises before and after the intergenerational succession, is the main parameter to be estimated in this part.

**Table 4:** Results of multiple difference in difference (DID).

Variable	FS			FR		
	Model 1	Model 2	Model 3	Model 4	Model 5	Model 6
Treatment × post	0.029^**^ [0.013]	0.028^**^ [0.013]	0.028^**^ [0.013]	0.004 [0.009]	0.004 [0.009]	0.003 [0.009]
Constant	0.005 [0.032]	0.308^*^ [0.185]	0.205 [0.323]	−0.083 [0.1529]	0.070 [0.174]	0.849^*^ [0.482]
Enterprise characteristics	NO	YES	YES	NO	YES	YES
Macro-economy	NO	NO	YES	NO	NO	YES
YEAR	YES	YES	YES	YES	YES	YES
INDIV	YES	YES	YES	YES	YES	YES
*N*	2,784					
*R* ^2^	0.1927	0.2075	0.2075	0.1168	0.1441	0.1441

* *p* < 0.1;

** *p* < 0.05.

Standard errors in brackets.

The columns of Model 1–3 in Table 4 estimate the level of assets financialization (FS) as the dependent variable. Model 1 is listed as the estimation result without the addition of control variables. Model 2 is listed as the estimation result with the addition of corporate characteristic control variables. Model 3 is listed as the estimation result with the addition of macroeconomic control variables. It is clear to see that the coefficient of Treatment × post is significantly positive (*p* < 0.05) whether the control variables are added or no, indicating the intergenerational succession has a significant positive impact on the asset financialization level of Chinese family enterprises. Therefore, Hypothesis 1 is supported. Moreover, the level of asset financialization of Chinese family enterprise increases by about 3% with intergenerational succession.

The columns of Model 4–6 of Table 4 estimate the level of revenue financialization (FR) as the dependent variable. It can be seen that the coefficient of Treatment × Post is not positive whether the control variables are added or not, indicating the intergenerational succession has no significant impact on the revenue financialization level of Chinese family enterprises (*p* > 0.1). Therefore, Hypothesis 2 is also supported.

Compared the different influence from intergenerational succession on FS and FR, it can be summarized that although the heirs of family enterprises have been effective in promoting the enterprises’ asset financialization level, such efforts have not effectively created new profits for the enterprises. It can be speculated that the aim of obtaining higher profits through financialization is not satisfactorily achieved, which is one of the important findings of this paper. Therefore, in addition to the existing research literature on the financialization of enterprises, a more comprehensive understanding of the relationship between intergenerational succession and the financialization of family enterprises has been obtained in this study. It is undoubtedly a major hidden danger for the long-term enterprise development when the heir inefficiently improves the asset financialization level of family enterprise.

### Robustness test

#### Placebo effect test

In this paper, the placebo effect was tested by replacing the treatment group and constructing the virtual intergenerational succession time. First, the treatment group was exchanged with the control group, and the intergenerational succession time for the exchanged treatment group was randomly set for DID analysis. If there was no placebo effect, the coefficient of Treatment × post of asset financialization level (FS) should not be significant after reestimation. The regression results are shown in Table 5, in the model with asset financialization level (FS) and revenue financialization level (FR) as dependent variables, the coefficients of Treatment × post and the key explanatory variable were not significant. These results demonstrate that the virtual treatment group and the intergenerational succession time have no influence on the financialization of family enterprises, which also prove that conclusions of this paper are robust to a certain extent.

**Table 5:** Results of placebo effect test.

Variable	FS			FR		
	Model 1	Model 2	Model 3	Model 4	Model 5	Model 6
Treatment × post	0.009 [0.014]	0.008 [0.014]	0.008 [0.014]	0.004 [0.010]	0.004 [0.010]	0.004 [0.010]
Constant	0.010 [0.042]	0.207 [0.283]	0.309 [0.334]	0.123 [0.153]	0.235 [0.354]	0.159 [0.184]
Enterprise characteristics	NO	YES	YES	NO	YES	YES
Macro-economy	NO	NO	YES	NO	NO	YES
Year	YES	YES	YES	YES	YES	YES
INDIV	YES	YES	YES	YES	YES	YES
N	2,784					
R2	0.155	0.178	0.178	0.155	0.162	0.162

Standard errors in brackets.

#### Extended matching range

In this paper, a total of 20,299 pieces of family enterprise data were obtained with the treatment group data of 1,392 pieces. The one-to-one matching samples accounted for about 13.7% of the total amount of data. Aiming to avoid possible underrepresentation due to the small sample size, the control group samples in the year-by-year PSM process was expanded by adopting the no placing back matching ratio of 1:2, 1:3, and 1:4 for further DID analysis, respectively. Part of the DID results (matching ratio of 1:2) are shown in Table 6. It is indicated that the research conclusions hold true in both the asset financialization model and revenue financialization model after the sample size was expanded.

**Table 6:** Analysis with DID method after expanding the sample size.

Variable	FS			FR		
	Model 1	Model 2	Model 3	Model 4	Model 5	Model 6
Treatment×Post	0.031^***^ [0.012]	0.031^***^ [0.011]	0.031^***^ [0.011]	0.013 [0.011]	0.010 [0.011]	0.010 [0.011]
Constant	−0.014 [0.033]	0.209 [0.135]	0.237 [0.251]	−0.160 [0.150]	0.908^*^ [0.487]	2.437 [1.518]
Enterprise characteristics	NO	YES	YES	NO	YES	YES
Macro-economy	NO	NO	YES	NO	NO	YES
YEAR	YES	YES	YES	YES	YES	YES
INDIV	YES	YES	YES	YES	YES	YES
*N*	4,048					
*R* ^2^	0.1445	0.1556	0.1556	0.1126	0.1202	0.1201

* *p* < 0.1;

*** *p* < 0.01.

Standard errors in brackets.

#### Narrowing the sample size

Since the global financial crisis in 2008, the resulting demand contraction and overcapacity had significant impacts on the normal revenue of Chinese family enterprises in the short term. In addition, the Sino-US trade war from 2018 and the outbreak of the global COVID-19 epidemic at the end of 2019 also had negative impacts on the long-term development strategy and confidence of Chinese family enterprises. These may give companies a greater incentive to avoid risk through financial asset allocation. In order to eliminate the bias caused by these major events, the enterprise data from 2009 to 2010 and 2019 to 2020 were removed. And then, the year-by-year PSM and multiple DID analyses were performed again, and the results are shown in Table 7. It can be seen that the coefficient of Treatment × post is still significantly positive (*p* < 0.1) in the model with FS as the dependent variable. Similarly, in the model where FR is the dependent variable, the coefficient of Treatment × post is still not significant. It is indicated that the research conclusion of this paper is still robust after removing the influence of potential extreme data.

**Table 7:** Analysis with DID method after narrowing the sample size.

Variable	FS			FR		
	Model 1	Model 2	Model 3	Model 4	Model 5	Model 6
Treatment×Post	0.026^*^ [0.015]	0.025^*^ [0.013]	0.025^*^ [0.013]	0.006 [0.012]	0.006 [0.012]	0.006 [0.012]
Constant	−0.016 [0.037]	0.231 [0.243]	0.387^*^ [0.268]	−0.250 [0.243]	−0.003 [0.353]	−0.041 [0.366]
Enterprise characteristics	NO	YES	YES	NO	YES	YES
Macro-economy	NO	NO	YES	NO	NO	YES
YEAR	YES	YES	YES	YES	YES	YES
INDIV	YES	YES	YES	YES	YES	YES
*N*	2,139					
*R* ^2^	0.178	0.196	0.196	0.152	0.172	0.172

* *p* < 0.1.

Standard errors in brackets.

### Further analysis of the heir’s growing experience

Based on the results above, we further examined the mechanism of intergenerational succession in the process of financialization of Chinese family enterprises from the perspective of heirs’ growing experience through the moderating effect test model. The summary of heirs’ typical growing experience and personal characteristics is shown in Table 8. Generally, the heirs of Chinese family enterprises have experienced an average of 9.73 years of in-company training before intergenerational succession. The average age for succession is 36 years old on average, and the average education level of heirs is between bachelor’s degree and master’s degree. In this paper, the information of heirs is collected and sorted manually, which is highly consistent with the description of other researchers (Cai et al., 2019; Zhu and Lv, 2019).

**Table 8:** Descriptive statistics of variables for heirs’ individual characteristics.

Variable	Obs	Mean	Std. Dev.	Min	Max
Childhood	1,392	0.429	0.495	0	1
Overseas	1,392	0.351	0.477	0	1
MBA	1,392	0.252	0.434	0	1
Outjob	1,392	0.574	0.495	0	1
Age	1,392	35.864	6.137	25	56
Cogdiff	1,392	1.418	1.208	−2	4
Train	1,392	9.730	4.931	1	30
Sex	1,392	0.839	0.368	0	1
Degree	1,392	3.577	1.295	1	5

Table 9 shows the test results of the moderating effect of heirs’ typical growth experience on the relation between the intergenerational succession and the financialization level of Chinese family enterprises. The regression result of FR as the dependent variable has been displayed in “Results for multiple DID” and was not significant. Thus, only the content related to FS is discussed here. In each model, the columns of “Model 1,” “Mode 3,” “Mode 5” and “Mode 7” represent regressive analysis controlling the company’s characteristic variables and macroeconomic variables, while columns of “Model 2,” “Model 4,” “Model 6” and “Model 8” represent that after further controlling the heirs’ individual characteristic variables. It can be found that the coefficient of Treatment × post is still significantly positive after adding various growing experiences of the heir, indicating that the growing experience of the heir plays a moderating effect.

**Table 9:** The moderating effect of heirs’ growing experiences.

FS	Childhood		Overseas		MBA		Outjob	
	Model 1	Model 2	Model 3	Model 4	Model 5	Model 6	Model 7	Model 8
Treatment × post	0.026^***^ [0.005]	0.030^***^ [0.006]	0.027^***^ [0.005]	0.033^***^ [0.006]	0.017^***^ [0.005]	0.022^***^ [0.006]	0.036^***^ [0.006]	0.042^***^ [0.007]
Treatment × post × childhood	0.020^***^ [0.007]	0.021^***^ [0.007]						
Treatment × post × overseas			0.019^**^ [0.008]	0.017^**^ [0.008]				
Treatment × post × MBA					0.051^***^ [0.008]	0.053^***^ [0.008]		
Treatment × post × outjob							−0.006 [0.007]	−0.008 [0.007]
Enterprise characteristics	YES	YES	YES	YES	YES	YES	YES	YES
Macro-economy	YES	YES	YES	YES	YES	YES	YES	YES
Heirs’ individual characteristics	NO	YES	NO	YES	NO	YES	NO	YES
YEAR	YES	YES	YES	YES	YES	YES	YES	YES
INDIV	YES	YES	YES	YES	YES	YES	YES	YES
*N*	2,784							
*R* ^2^	0.148	0.150	0.147	0.149	0.160	0.162	0.146	0.148

** *p* < 0.05;

*** *p* < 0.01.

Standard errors in brackets.

The moderating effect of the parents’ entrepreneurial experience in the heir’s childhood is significantly valued at the level of 0.01, supporting Hypothesis 3. This indicates that the heirs who have witnessed their parents’ entrepreneurial process during their childhood are more inclined to improve the asset financialization level of the enterprise after taking over the family business. This might be attributed to the experience of the hardship of their parents’ entrepreneurship which weakens the motivation and tendency of the heirs to “keep the business” to a certain extent. The rich growing environment and the family relationship spending more time apart than together may make the heirs more resistant to devote themselves into the family enterprises development as committed as their parents, which has been verified in the previous report (Cai et al., 2019).

The moderating effect of heirs’ overseas study experience is significantly valued at the level of 0.05, therefore Hypothesis 4 is supported. This indicates that heirs with overseas study experience tend to improve enterprise performance through allocation of financial assets after inheriting the management power, although the result of the management strategy is not so ideal, which is consistent with the logic of Zhu and Lv’s (2019). More open international vision as well as more modern management philosophy make the heirs divergent from their parents on ideas and visions. On the other hand, it is harder for the heirs who study overseas to obtain those intangible resources of their parents, which making it more difficult to run a business on the original way. It may be the reason and the intrinsic motivation why the heirs seek a different approach in the field of financialization.

The moderating effect of heirs’ MBA education experience is significantly valued at the level of 0.01, and Hypothesis 5 is supported. It is indicated that heirs with MBA education experience are more likely to promote the financialization process of family enterprises, which is consistent with the results of Li et al. (2016) and Cai et al. (2019) on the relationship between intergenerational succession and family enterprise’ strategic adjustment. The heir who took MBA education tend to regard the enterprise more as a pure profit “enterprise,” rather than a “career” with the meaning of spiritual inheritance. Meanwhile, the MBA experience gives the heir more chance to communicate with other entrepreneurs and get more business information, which provides them more convenience when they consider changing the enterprise original direction.

The moderating effect of the heirs’ working experience outside the family enterprise is not significant (*p* > 0.1), therefore the hypothesis 6 is not supported. The possible reason may be that most heirs have working experience outside the family enterprise in the sample range selected in this paper, resulting in insufficient differentiation of this variable. It may also be due to the lack of complete and detailed information about the heir’s out-of-enterprise work experience, meanwhile the dichotomous variable used in this paper makes it impossible to measure the potential impact of the time-period and industry of out-of-enterprise work experience.

## Discussion

The current study explored the influence from the intergenerational succession on the financialization of Chinese family enterprises and the role of the heirs’ typical growing experience in it with a mixed-methods of economics and psychology. The results showed that intergenerational succession had a significantly positive impact on the asset financialization of Chinese family enterprises, but not on the revenue financialization. It meant that heirs’ financialization behavior had not brought about effective financial profits. The results also showed that heirs who had witnessed their parents’ entrepreneurial experience in their childhood or had overseas study experience or MBA education experience were more inclined to promote asset financialization after taking over the family business, while outside working experience in other companies had no significant effect. The growing experience of heirs were important moderating factor for the relationship between intergenerational succession and financialization of Chinese family enterprises.

As mentioned above, FS represented the behavior of heirs advancing the financialization of the enterprises, and FR represented the outcome of that behavior. The significant increase in the level of asset financialization verified that the crowding-out effect was confirmed, indicating that heirs tried to make profits by increasing financial investment. It is worth considering why the level of revenue financialization has not increased significantly. The crowding-out effect made the main business investment of enterprises be replaced, which might lead to the relative decline of the main business income. However, the proportion of financial income had not increased significantly, indicating that the financialization promoted by heirs might be even counterproductive. Further combining with the moderating effect of their growth experiences, the first three kinds of experiences seemed to only promote the successor’s financialization behavior at the psychological level but could not guarantee the outcome of their behavior. Given the complexity and less-than-ideal state of China’s financial market, it is not enough for more skilled participants to outperform, not to mention confident and risk-prone heirs who lack financial expertise. In short, financialization is not a good choice for Chinese family enterprises in the process of intergenerational succession. If we further consider its possible impact on China’s macro economy, it is even more alarming.

In summary, the findings in this study are theoretically valuable in the following three aspects. Firstly, this paper expanded the topic of family enterprise research. Based on the theoretical derivation, this paper empirically tested the relationship between generational succession and financialization of Chinese family enterprises. This is of positive significance for understanding their current business situation. Secondly, a new way was applied to examine the financialization of enterprises. FS and FR were used to examine the behavior and outcome of heirs’ financialization, supplying valuable references for other studies. Thirdly, we explored the psychological motivation of heirs to promote financialization. Starting from the typical growth experience of heirs, this paper explored the deep reasons behind the promotion of financialization behavior by heirs at the psychological development level, providing an interdisciplinary perspective.

Meanwhile, this paper still has the following three deficiencies. Firstly, considering the availability and completeness, the data used in this paper are all from listed companies. However, listed companies only account for a part of China’s family enterprises, and they play the role of shadow banking fund supplier to some extent, which may lead to the deviation between FS and FR calculated based on their financial statement data. Secondly, to obtain continuous and complete financial asset information, this paper intercepts the data of listed companies since 2009. However, due to the impact of the 2008 global financial crisis, both the global economic environment and China’s macroeconomic conditions have undergone significant changes, which may lead to the deviation of the financialization behavior of the selected enterprises from that before 2008. Thirdly, the personal characteristics data of heirs used in this paper are all from the collection and collation of public information. To ensure the accuracy of information, we have to sacrifice some precision. For example, if the successor’s work experience outside the enterprise can be divided more carefully, we may obtain more valuable research results. The above deficiencies need to be further expanded and improved. The intergenerational succession of Chinese family enterprises is still in its rapid progress. Many new challenges and influencing factors are emerging. In future, more refined and diversified data on heirs and family enterprises could be further collected for thoroughly investigating the relationship between the two variables and the moderating effects of heirs’ growth experience.

## Data availability statement

The original contributions presented in the study are included in the article/supplementary material, further inquiries can be directed to the corresponding authors.

## Author contributions

XZ and HW conceived and designed the research and provided guidance throughout the entire research process. SY wrote the main part of the manuscript. WW and YL collected the data and wrote the methods section. All authors contributed to the article and approved the submitted version.

## Funding

This work was supported in part grants from Major Supporting Project of the National Social Science Fund of China (21BJY002 and 20AZD033) and The Philosophy and Social Sciences in Zhejiang Province (20XXJC03ZD).

## Conflict of interest

The authors declare that the research was conducted in the absence of any commercial or financial relationships that could be construed as a potential conflict of interest.

## Publisher’s note

All claims expressed in this article are solely those of the authors and do not necessarily represent those of their affiliated organizations, or those of the publisher, the editors and the reviewers. Any product that may be evaluated in this article, or claim that may be made by its manufacturer, is not guaranteed or endorsed by the publisher.

## References

[ref1] Ahrens J. P. Calabrò A. Huybrechts J. Woywode M. (2019). The enigma of the family successor–firm performance relationship: a methodological reflection and reconciliation attempt. Entrep. Theory Pract. 43, 437–474. doi: 10.1177/1042258718816290

[ref2] Anderson R. C. Duru A. Reeb D. M. (2012). Investment policy in family-controlled firms. J. Bank. Financ. 36, 1744–1758. doi: 10.1016/j.jbankfin.2012.01.018

[ref3] Bai X. O. Tsang E. W. K. Xia W. (2020). Domestic versus foreign listing: does a CEO’s educational experience matter?. J. Bus. Ventur. 35:105906. doi: 10.1016/j.jbusvent.2018.10.004

[ref4] Bennedsen M. Nielsen K. Pérez-González F. Wolfenzon D. (2005a). Inside the family firm: the role of families in succession decisions and performance. Q. J. Econ. 122, 647–691. doi: 10.1162/qjec.122.2.647

[ref200] Bennedsen M. Nielsen K. M. Wolfenzon D. (2005b). The family behind the family firm: Evidence from successions in Danish firms. NYU Working Paper No.:CLB-06-002.

[ref300] Bertrand M. Johnson S. Samphantharak K. Schoar A. (2008). Mixing Family with Business: A Study of Thai Business Groups and the Families behind Them. J. Financ. Econ. 88, 466–498. doi: 10.1.1.570.5528

[ref5] Bernile G. Bhagwat V. Rau P. R. (2017). What doesn’t kill you will only make you more risk-loving: early-life disasters and CEO behavior. J. Financ. 72, 167–206. doi: 10.1111/jofi.12432

[ref6] Bin-Feng C. Mirza S. S. Ahsan T. Safdar R. Iqbal A. Hayat M. (2022). Family control and corporate risk-taking in China: does working capital strategy matter?. Leprosy–Botany: jatropha gossypifolia, anti-leprous shrub 35, 4280–4299. doi: 10.1080/1331677X.2021.2013270

[ref7] Cai Q. Chen Y. Wu J. (2019). Growth experience of the second generation and M&a behavior in family firms: evidence from China. Nankai Bus. Rev. 1, 139–150.

[ref8] Carr J. C. Sequeira J. M. (2007). Prior family business exposure as intergenerational influence and entrepreneurial intent: a theory of planned behavior approach. J. Bus. Res. 60, 1090–1098. doi: 10.1016/j.jbusres.2006.12.016

[ref9] Chen C. (2018). Intergenerational succession of family firms: a continuation or rupture of the innovation spirit? Manag. Rev. 30, 81–92. doi: 10.14120/j.cnki.cn11-5057/f.2018.06.007

[ref10] Chen M. Xiao J. Z. Zhao Y. (2021). Confucianism, successor choice, and firm performance in family firms: evidence from China. J. Corp. Finan. 69:102023. doi: 10.1016/j.jcorpfin.2021.102023

[ref11] Cucculelli M. (2009). Owner identity and firm performance: evidence from European companies. Marco Cucculelli 98, 149–178.

[ref12] Custódio C. Metzger D. (2014). Financial expert CEOs: CEOs work experience and firm’s financial policies. J. Financ. Econ. 114, 125–154. doi: 10.1016/j.jfineco.2014.06.002

[ref13] Dai W. Liu Y. Liao M. (2016). The imprinting of Danwei system and “making fast bucks”: evidence from China’s private enterprises. Manage. World 2016, 99–115–187–188.

[ref600] Davis P. S. Harveston P. D. (2001). The phenomenon of substantive conflict in the family firm: a cross-generational study. J. Small Bus. Manag. 39, 14–30. doi: 10.1111/0447-2778.00003

[ref14] Dehejia R. H. Wahba S. (1998). Propensity score-matching methods for nonexperimental causal studies. Rev. Econ. Stat. 84, 151–161.

[ref15] Du Y. Xie J. Chen J. Y. (2019). CEO’s financial background and the financialization of entity enterprises. China Industrial Econ. 8, 136–154. doi: 10.19581/j.cnki.ciejournal.2019.05.008

[ref16] Du Y. Zhang H. Chen J. Y. (2017). The impact of financialization on future development of real enterprises’ core business: promotion or inhibition. China Industrial Econ. 14, 113–131. doi: 10.19581/j.cnki.ciejournal.20171214.007

[ref17] Duchin R. Ozbas O. Sensoy B. A. (2009). Costly external finance, corporate investment, and the subprime mortgage credit crisis. New York, NY: Social Science Electronic Publishing, 97,418–435.

[ref18] Faleye O. Kovacs T. Venkateswaran A. (2014). Do better-connected CEOs innovate more? J. Financ. Quant. Anal. 49, 1201–1225. doi: 10.1017/S0022109014000714

[ref19] Feng Y. Yao S. Wang C. Liao J. Cheng F. (2022). Diversification and financialization of non-financial corporations: evidence from China. Emerg. Mark. Rev. 50:100834. doi: 10.1016/j.ememar.2021.100834

[ref20] Giannetti M. Liao G. Yu X. (2015). The brain gain of corporate boards: evidence from China. J. Financ. 70, 1629–1682. doi: 10.1111/jofi.12198

[ref800] Greenaway D. Gullstrand J. Kneller R. (2005). Exporting May Not Always Boost Firm Productivity. Rev. World Econ. 141, 561–582. doi: 10.1007/s10290-005-0045-5

[ref500] Goldberg S. D. Wooldridge B. (1993). Self-confidence and managerial autonomy: Successor characteristics critical to succession in family firms. Family Bus. Rev. 6, 55–73. doi: 10.1111/j.1741-6248.1993.00055

[ref21] Hambrick D. C. Nadler D. A. Tushman M. L. (1998). Navigating Change: How the CEOs, Top Teams and Boards Steer Transformation[M]. Boston, MA: Harvard Business School Press.

[ref900] Heyman F. Sjöholm F. Tingvall P. G. (2007). Is there really a foreign ownership wage premium? Evidence from matched employer–employee data. J. Int. Econ. 73, 355–376. doi: 10.1016/j.jinteco.2007.04.003

[ref22] Hu Y. Wang X. Zhang J. (2017). The motivation for financial asset allocation: reservoir or substitution?: evidence from Chinese listed companies. Econ. Res. J. 52, 181–194.

[ref23] Hu X. Wu Y. (2016). Study on transgenerational transfer of political Capital of Chinese Family Firm——an Empirical Analysis Based on entrepreneurs’ membership of PC or CPPCC. China Industrial Econ. 1, 146–160. doi: 10.19581/j.cnki.ciejournal.2016.01.010

[ref24] Huang H. Lu C. Zhu X. (2018). Second generation involvement and corporate innovation: evidence from China. Nankai Bus. Rev. 10, 526–545. doi: 10.1108/NBRI-03-2019-0008

[ref25] Ishfaq M. Nazir M. S. Qamar M. A. J. Usman M. (2020). Cognitive bias and the extraversion personality shaping the behavior of investors. Front. Psychol. 11:556506. doi: 10.3389/fpsyg.2020.556506, PMID: 33178066 PMC7593711

[ref26] Ke Y. Li Y. Wu X. (2019). Controlling Shareholder’s equity pledge and corporate investment——from the perspective of financial investment and real investment. Fin. Trade Econ. 40, 50–66. doi: 10.19616/j.cnki.bmj.2019.12.008

[ref27] Khan T. M. Bai G. Fareed Z. Quresh S. Khalid Z. Khan W. A. (2021). CEO tenure, CEO compensation, corporate social and environmental performance in China: the moderating role of coastal and non-coastal areas. Front. Psychol. 11:574062. doi: 10.3389/fpsyg.2020.574062 PMC7862113 33551900

[ref28] Krippner G. R. (2005). The financialization of the American economy. Soc. Econ. Rev. 3, 173–208. doi: 10.1093/SER/mwi008

[ref29] Leve L. D. Neiderhiser J. M. Scaramella L. V. (2010). The early growth and development study: using the prospective adoption design to examine genotype-environment interplay. Behav. Genet. 40, 306–314. doi: 10.1007/s10519-010-9353-1, PMID: 20358398 PMC5575997

[ref30] Levin D. Z. Barnard H. (2013). Connections to distant knowledge: interpersonal ties between more-and less-developed countries. J. Int. Bus. Stud. 44, 676–698. doi: 10.1057/jibs.2013.28

[ref31] Li X. Han J. Li W. (2015). Is it succession of the family business or creating other field: the construction of the authority legitimacy of the second-generation succession of the family business. J. Manage. World 6, 110–124, 187-188.

[ref1000] Li X. Zhang P. Ye W. (2016). Cognitive differences of the second generation of family and the adjustment of corporate diversification strategy: an empirical study based on the entry sample of the second generation of listed family firms in China. Journal of Sun Yatsen University (Social Science Edition) 03, 183–193. doi: 10.13471/j.cnki.jsysusse.2016.03.019

[ref32] Li H. Hang Y. Shah S. G. M. Akram A. Ozturk I. (2020). Demonstrating the impact of cognitive CEO on firms’ performance and CSR activity. Front. Psychol. 11:278. doi: 10.3389/fpsyg.2020.00278, PMID: 32184747 PMC7058782

[ref33] Li X. He X. Zou L. (2021). Family business research: theoretical Progress and future directions. J. Manag. World 11, 207–228. doi: 10.19744/j.cnki.11-1235/f.2020.0178

[ref34] Li W. Xu S. Li W. (2021). Growth experiences of the second generation and portfolio entrepreneurship in family firms: insights from the imprinting theory. Foreign Econ. Manag. 43, 126–140. doi: 10.16538/j.cnki.fem.20201229.401

[ref35] Liu X. Su C. Shao H. (2021). Intergenerational succession and excess control of family board seats. Econ. Res. J. 56, 111–129.

[ref36] Malmendier U. Tate G. Yan J. (2011). Overconfidence and early-life experiences: the effect of managerial traits on corporate financial policies. J. Financ. 66, 1687–1733. doi: 10.1111/j.1540-6261.2011.01685.x

[ref37] Marquis C. Tilcsik A. (2013). Imprinting: toward a multilevel theory. New York, NY: Social Science Electronic Publishing, 195–245.

[ref38] Miller D. Xu X. (2016). A fleeting glory: self-serving behavior among celebrated MBA CEOs. J. Manag. Inq. 25, 286–300. doi: 10.1177/1056492615607975

[ref39] Miroshnychenko I. de Massis A. Miller D. Barontini R. (2021). Family business growth around the world. Entrep. Theory Pract. 45, 682–708. doi: 10.1177/1042258720913028

[ref40] O’Sullivan D. Zolotoy L. Fan Q. (2021). CEO early-life disaster experience and corporate social performance. Strateg. Manag. J. 42, 2137–2161. doi: 10.1002/smj.3293

[ref41] Pan Y. Weng R. Xu N. (2018). The role of corporate philanthropy in family firm succession: a social outreach perspective. J. Bank. Financ. 88, 423–441. doi: 10.1016/j.jbankfin.2018.01.011

[ref42] Peng Y. C. Han X. Jian-Jun L. I. (2018). Economic policy uncertainty and corporate financialization. China Industrial Econ. 15, 137–155. doi: 10.19581/j.cnki.ciejournal.20180115.010

[ref43] Penman S. H. Yehuda N. (2009). The pricing of earnings and cash flows and an affirmation of accrual accounting. Rev. Acc. Stud. 14, 453–479. doi: 10.1007/s11142-009-9109-4

[ref44] Pieper T. M. Smith A. D. Kudlats J. (2015). The persistence of multifamily firms: founder imprinting, simple rules, and monitoring processes. Entrep. Theory Pract. 39, 1313–1337. doi: 10.1111/etap.12179

[ref45] Porfírio J. A. Felício J. A. Carrilho T. (2020). Family business succession: analysis of the drivers of success based on entrepreneurship theory. J. Bus. Res. 115, 250–257. doi: 10.1016/j.jbusres.2019.11.054

[ref46] Qi Y. Yang Y. Yang S. Lyu S. (2021). Does government funding promote or inhibit the financialization of manufacturing enterprises? Evidence from listed Chinese enterprises. North Am. J. Econ. Finance 58:101463. doi: 10.1016/j.najef.2021.101463

[ref100] Sarfraz M. Shah S. G. M. Ivascu L. Qureshi M. A. A. (2022). Explicating the impact of hierarchical CEO succession on small-medium enterprises’ performance and cash holdings. Int. J. Finance Econ. 27, 2600–2614. doi: 10.1002/ijfe.2289

[ref47] Song J. Yang L. (2015). U-shape relationship between non-currency financial assets and operating profit: evidence from Financialization of Chinese listed non-financial corporates. J. Financ. Res. 6, 111–127.

[ref48] Su K. Liu H. (2021). Financialization of manufacturing companies and corporate innovation: lessons from an emerging economy. Manag. Decis. Econ. 42, 863–875. doi: 10.1002/mde.3278

[ref49] Tang H. Zhang C. (2019). Investment risk, return gap, and financialization of non-listed non-financial firms in China. Pac. Basin Financ. J. 58:101213. doi: 10.1016/j.pacfin.2019.101213

[ref400] Villalonga B. Amit R. (2006). How do family ownership, control and management affect firm value? J. Financ. Econ. 80, 385–417. doi: 10.1016/j.jfineco.2004.12.005

[ref50] Wang G. (2018). The internal mechanism of finance transfer from the real economy to the fictitious economy and deepening supply-side structure reform. China Industrial Econ. 11, 5–23. doi: 10.19581/j.cnki.ciejournal.2018.07.001

[ref51] Wang H. J. Cao Y. Yang Q. (2017). Does the financialization of non-financial enterprises promote or inhibit corporate innovation. Nankai Bus. Rev 20, 155–166. doi: 10.3969/j.issn.1008-3448.2017.01.014

[ref52] Wang Y. Liang G. Wang H. (2021). Generation and iteration of successor’s entrepreneurial schema in family firms: multiple case study based on imprinting theory. J. Manag. World 37, 198–216. doi: 10.19744/j.cnki.11-1235/f.2021.0057

[ref53] Weng T. C. Chi H. Y. (2019). Family succession and business diversification: evidence from China. Pac. Basin Financ. J. 53, 56–81. doi: 10.1016/j.pacfin.2018.09.001

[ref54] Wu Y. Li S. Cao Z. (2021). The effect of financialization on management earnings forecasts. Appl. Econ. Lett. 16, 1–4.

[ref55] Wyrwich M. (2015). Entrepreneurship and the intergenerational transmission of values. Small Bus. Econ. 45, 191–213. doi: 10.1007/s11187-015-9649-x

[ref56] Xiong R. Wei P. Yang J. Cristofini L. A. (2021). Impact of top executive turnover on firms’ R&D investment: evidence from China. Innovations 23, 400–424. doi: 10.1080/14479338.2020.1836968

[ref57] Xu N. Xie R. Wu S. (2019). Chinese–style family business management: governance models, leadership model and firm performance. Econ. Res. J. 54, 165–181.

[ref58] Xu X. Xuan C. (2021). A study on the motivation of financialization in emerging markets: the case of Chinese nonfinancial corporations. Int. Rev. Econ. Finance 72, 606–623. doi: 10.1016/j.iref.2020.12.026

[ref59] Xun H. Tian G. Li J. (2017). Shadow banking business and financial structure of non-financing enterprises—empirical evidence from Chinese listed companies. Stud. Int. Finance 10, 44–54. doi: 10.16475/j.cnki.1006-1029.2017.10.005

[ref60] Yan H. Chen B. (2018). The financial assets of industrial listed corporations: the market effect and holding motivation. Econ. Res. J. 53, 152–166.

[ref61] Yang B. Nahm A. Song Z. (2022). Succession, political resources, and innovation investments of family businesses: evidence from China. Manag. Decis. Econ. 43, 321–338. doi: 10.1002/mde.3385

[ref700] Yang Z. Wang H. J. Dai J. Xu C. H. (2019). Deregulation of interest rates, equalization of profit-rate, and enterprises’ shift from real to virtual. J. Financ. Res. 468, 20–38.

[ref62] Yu L. Zhao D. Xue Z. Gao Y. (2020). Research on the use of digital finance and the adoption of green control techniques by family farms in China. Technol. Soc. 62:101323. doi: 10.1016/j.techsoc.2020.101323

[ref63] Zhang C. Luo L. (2021). Board diversity and risk-taking of family firms: evidence from China. Int. Entrep. Manag. J. 17, 1569–1590. doi: 10.1007/s11365-021-00769-z

[ref64] Zhang C. Zhang B. (2016). The falling real investment puzzle: a view from financialization. Econ. Res. J. 51, 32–46.

[ref65] Zhao Y. Su K. (2022). Economic policy uncertainty and corporate financialization: evidence from China. Int. Rev. Financ. Anal. 82:102182. doi: 10.1016/j.irfa.2022.102182

[ref66] Zhu Z. Li X. Zhao Y. (2021). Co-governance and innovation decision: paternalism and foresight effect in the intergenerational succession of Chinese family firm. J. Manage. World 37, 191–206–232+207. doi: 10.19744/j.cnki.11-1235/f.2021.0146

[ref67] Zhu X. Lv C. (2019). Cultivating successors of the family firm: overseas training or domestic training. Econ. Res. J. 54, 68–84.

